# A Predictive Model for Perinatal Brain Injury Using Machine Learning Based on Early Birth Data

**DOI:** 10.3390/children11111313

**Published:** 2024-10-29

**Authors:** Ga Won Jeon, Yeong Seok Lee, Won-Ho Hahn, Yong Hoon Jun

**Affiliations:** Department of Pediatrics, Inha University Hospital, Inha University College of Medicine, Incheon 22332, Republic of Korea; iamgawon@inha.ac.kr (G.W.J.); yslee89@inhauh.com (Y.S.L.); neonatology@inha.ac.kr (W.-H.H.)

**Keywords:** machine learning, infant, hypoxic ischemic encephalopathy, therapeutic hypothermia, magnetic resonance imaging

## Abstract

Background/Objective: It is difficult to predict perinatal brain injury, and performing brain magnetic resonance imaging (MRI) based on suspected injury remains a clinical challenge. Therefore, we aimed to develop a reliable method for predicting perinatal brain injury using a machine learning model with early birth data. Methods: Neonates admitted to our institution from January 2017 to June 2024 with a gestational age of ≥36 weeks, a birth weight of ≥1800 g, admission within 6 h of birth, and who underwent brain MRI to confirm perinatal brain injury were included. Various machine learning models, including gradient boosting, were trained using early birth data to predict perinatal brain injury. Synthetic minority over-sampling and adaptive synthetic sampling (ADASYN) were applied to address class imbalance. Model performance was evaluated using accuracy, F1 score, and ROC curves. Feature importance scores and Shapley additive explanations (SHAP) values were also calculated. Results: Among 179 neonates, 39 had perinatal brain injury. There were significant differences between the injury and non-injury groups in mode of delivery, Apgar scores, capillary pH, lactate dehydrogenase (LDH) levels, and whether therapeutic hypothermia was performed. The gradient boosting model with the ADASYN method achieved the best performance. In terms of feature importance scores, the 1 min Apgar score was the most influential predictor. Additionally, SHAP analysis showed that LDH levels had the highest SHAP values. Conclusion: the gradient boosting model with ADASYN oversampling effectively predicts perinatal brain injury, potentially improving early detection for predicting long-term outcomes, reducing unnecessary MRI scans, and lowering healthcare costs.

## 1. Introduction

Perinatal brain injury represents a significant clinical challenge in both high-income and low-resource countries among term neonates [1]. This condition affects approximately 1 to 3 per 1000 term infants, with hypoxic-ischemic encephalopathy (HIE) being the most common cause [2,3]. HIE results from a hypoxic-ischemic event during the prenatal, intrapartum, or postnatal period, leading to inadequate blood flow to the infant’s brain and subsequent brain injury [4]. This deprivation initiates a cascade of pathophysiological mechanisms, including excitotoxicity, apoptosis, inflammation, and subacute injury to white and grey matter, which ultimately leads to prolonged brain atrophy [5]. HIE is associated with various neuropsychological, cognitive, and psychopathological complications that may continue into adolescence, as demonstrated by the higher incidence of cognitive impairments and mental health disorders in affected individuals [6]. The severity of HIE is defined by clinical findings and is used to select infants for therapeutic hypothermia, which mitigates the effects of HIE by reducing free radical production, lowering glutamate levels, decreasing oxygen demand, and minimizing apoptosis [7,8]. Hypothermia treatment has also been demonstrated to significantly lower the risks of mortality and morbidity [9,10,11]. However, previous studies indicate that the long-term effects of therapeutic hypothermia remain inconclusive, as there is a lack of comprehensive data regarding its influence on neurodevelopmental outcomes [12]. Perinatal brain injury can also be caused by perinatal stroke, which occurs in approximately 1 per 1000 live births [13], with the highest risk occurring around the time of birth [14,15]. Perinatal stroke has been associated with long-term cognitive challenges, including poorer academic performance and reduced receptive and expressive language skills [16]. When perinatal brain injury is suspected, brain magnetic resonance imaging (MRI) is crucial for diagnosis and predicting long-term outcomes. However, predicting perinatal brain injury to perform brain MRI is challenging due to the nonspecific nature of its clinical manifestations [17]. Moreover, in some instances, brain MRI reveals evidence of brain injury due to HIE, even though therapeutic hypothermia was not initiated at birth, as the treatment criteria were not met at that time. Currently, no reliable method exists for predicting perinatal brain injury, and selecting neonates for performing brain MRI based on suspected injury is a considerable clinical challenge. Therefore, we aimed to use a machine learning model with early birth data to develop a reliable method for predicting perinatal brain injury.

## 2. Related Work

This study represents the first attempt to predict perinatal brain injury using machine learning approaches, although several prior investigations employed machine learning models to predict various neonatal diseases. A prior study utilized data from the Korean Neonatal Network, a nationwide cohort registry of preterm infants in Korea, to develop machine learning models for predicting severe intraventricular hemorrhage or early mortality in very-low-birth-weight infants weighing less than 1500 g. The models were constructed using logistic regression with ridge regularization, random forest, and extreme gradient boosting (XGB) algorithms. Among these, the XGB model demonstrated superior performance, achieving an accuracy of 0.90, with predictions based on clinical parameters collected within the first week of life [18]. Similarly, another study employed the Korean Neonatal Network dataset to develop predictive models for the early onset of surgical necrotizing enterocolitis (NEC) in neonates. An ensemble model was developed, achieving an area under the receiver operating characteristic curve (AUC) of 0.721, with the authors suggesting that this model could facilitate the early identification of neonates at increased risk of requiring surgical intervention for NEC [19]. Additionally, a study introduced a stacking model for the early detection and accurate diagnosis of neonatal sepsis, birth asphyxia, NEC, and respiratory distress syndrome, reporting an accuracy of 97.04% [20]. Another study demonstrated that machine learning models, including artificial neural networks (ANN), decision trees, support vector machines (SVC), Bayesian networks, and ensemble models, can effectively predict neonatal mortality in the neonatal intensive care unit (NICU) [21]. Efforts to predict adverse birth outcomes in very-low-birth-weight infants have also been made. One study utilized machine learning models, including artificial neural networks (ANN), decision trees, logistic regression, naïve Bayes, random forests, and SVC, to analyze key predictors of adverse outcomes such as particulate matter concentration (PM10) in a Korean Neonatal Network dataset. Among the six models, the random forest exhibited the best performance, with an accuracy of 0.79 and an area under the receiver operating characteristic curve (AUC) of 0.72 [22].

## 3. Materials and Methods

### 3.1. Participants and Sample Collection

We assessed the medical records of patients admitted to the NICU at Inha University Hospital between January 2017 and June 2024. The inclusion criteria consisted of a gestational age greater than 36 weeks, a birth weight of more than 1800 g, admission within 6 h of birth for early postnatal data collection, and the performance of brain MRI during hospitalization. We excluded neonates who underwent brain MRI for the workup of congenital anomalies of the central nervous system. The initial data used to predict perinatal brain injury with machine learning included the following variables (Table 1). Perinatal brain injury was defined as the presence of HIE, perinatal arterial ischemic stroke, intracranial hemorrhage, or cerebral sinovenous thrombosis were confirmed on a subsequent brain MRI.

### 3.2. Statistical Analysis

The characteristics of the no brain injury group and the brain injury group were compared. Continuous variables that followed a normal distribution were analyzed using the Student’s *t*-test, whereas those that did not follow a normal distribution were examined using the Wilcoxon rank sum test. Categorical variables were assessed using the χ^2^ test, Fisher’s exact test, or linear-by-linear association for comparisons between groups. All statistical analyses were performed using SPSS 26 software (version 26.0, SAS Institute, Cary, NC, USA).

### 3.3. Model Selection and Hyperparameter Tuning

To conduct supervised learning, we split the dataset into training and testing sets with an 80:20 ratio. Subsequently, we trained the following models: SVC, random forest, k-nearest neighbors (KNN), Gaussian naïve Bayes, gradient boosting, decision tree, AdaBoost, extra trees, and easyensemble. For each model, we tuned the relevant hyperparameters using grid search with cross-validation to find the optimal settings.

### 3.4. Imbalanced Datasets Processing

In our dataset, the number of patients confirmed with perinatal brain injury on MRI is significantly smaller compared to those without the condition. Most machine learning algorithms tend to perform poorly on the minority class due to this class imbalance. To address the class imbalance in the target variable, we employed two oversampling techniques: synthetic minority over-sampling technique (SMOTE) and adaptive synthetic sampling (ADASYN).

### 3.5. Hyperparameter Tuning and Cross-Validation

Hyperparameter tuning process was performed to optimize the performance of various machine learning classifiers. The following classifiers and their respective parameter grids were utilized:

(1) SVC: the regularization parameter was set to a range from 0.1 to 1, the gamma parameter was set to a range from 0.001 to 0.01, and the kernel function was limited to ‘linear’. (2) Random forest: the number of trees in the forest was set to a range from 50 to 100, and the maximum depth of each tree was set to a range from 10 to 20. (3) KNN: the number of neighbors was set to a range from 3 to 5, with weight options as [‘uniform’, ‘distance’]. (4) Gradient boosting: the number of boosting stages was set to a range from 50 to 100, the learning rate was set to a range from 0.01 to 0.1, and the maximum depth of the individual estimators was set to a range from 3 to 5. (5) Decision tree: parameters included the maximum depth of the tree with values set to a range from 10 to 20, the minimum samples for splitting an internal node set to a range from 2 to 5, and the minimum samples required to be at a leaf node set to a range from 1 to 2. (6) AdaBoost: this classifier had the number of boosting stages set to a range from 50 to 100 and the learning rate set to a range from 0.01 to 0.1. (7) Extra trees: the parameter grid included the number of trees in the forest set to a range from 50 to 100, the maximum depth of the trees set to a range from 10 to 20, the minimum samples for splitting an internal node set to a range from 2 to 5, and the minimum samples required to be at a leaf node set to a range from 1 to 2. (8) Gaussian naïve Bayes and easyensemble: default parameters were used, as no specific grid was defined.

A 5-fold cross-validation approach was utilized to evaluate the performance of each classifier.

### 3.6. Performance Evaluation

We assessed the accuracy, F1 score, and ROC curve for each model. To elucidate the decision-making process of the model, feature importance scores were computed for the optimal model, and the top 15 most influential features were visualized using a bar chart. Shapley additive explanations (SHAP) values were computed to assess the impact of each feature on the model’s predictions [24]. A bar plot of SHAP values was created to display the mean absolute SHAP values for the 15 most influential contributing features. All performance evaluations were carried out using Python (version 3.12).

## 4. Results

### 4.1. Demographics of Patients

A total of 179 neonates were initially enrolled in the study, with 3 subsequently excluded. Among the included patients, 39 were in the brain injury group, identified by brain MRI performed during hospitalization, while 137 were in the no brain injury group. When comparing the characteristics of the two groups, the brain injury group had a significantly higher rate of vaginal delivery and hypothermia treatment. Additionally, the 1 min and 5 min Apgar scores were significantly lower in the brain injury group, with lower capillary pH and higher LDH levels. Conversely, no significant differences were observed between the two groups with respect to sex, gestational age, birth weight, intrapartum complications, or neonatal symptoms, as well as blood pressure, CO_2_ levels, base excess, hemoglobin, platelet count, uric acid, albumin, mode of ventilation, fraction of inspired oxygen (FiO_2_), or comorbidities such as transient tachypnea of the newborn, meconium aspiration syndrome, pneumothorax, respiratory distress syndrome, and persistent pulmonary hypertension of the newborn (Table 2).

### 4.2. Performance Evaluation Based on Models and Oversampling Techniques

Various models were used with oversampling techniques to predict brain injury confirmed by brain MRI using data from within the first 6 h after birth. For the SVC model utilizing SMOTE oversampling, the optimal performance was achieved with a regularization parameter of 1 and a kernel coefficient of 0.001, resulting in an accuracy of 0.72 and an F1 score of 0.54. The random forest model performed best with a maximum tree depth of 20 and 50 trees, yielding an accuracy of 0.69 and an F1 score of 0.15. In the KNN model, the optimal configuration used five nearest neighbors and equal weighting for all neighbors, producing an accuracy of 0.64 and an F1 score of 0.52. The naïve Bayes model demonstrated an accuracy of 0.50 and an F1 score of 0.44. The gradient boosting model performed optimally with a learning rate of 0.01 and 100 boosting stages, achieving an accuracy of 0.72 and an F1 score of 0.58. The decision tree model, with a maximum tree depth of 10 and a minimum of two samples for node splitting, resulted in an accuracy of 0.56 and an F1 score of 0.27. The AdaBoost model, with a learning rate of 0.01 and 100 weak learners, demonstrated an accuracy of 0.67 and an F1 score of 0.57. In the extra trees model, optimal performance was obtained with a maximum tree depth of 20 and a minimum of two samples per leaf node, producing an accuracy of 0.69 and an F1 score of 0.15. Finally, the easyensemble model achieved an accuracy of 0.72 and an F1 score of 0.44. Overall, the gradient boosting model exhibited the highest accuracy and F1 score among all models (Table 3).

The models were also assessed using the ADASYN oversampling technique. For the SVC model, the optimal hyperparameters were identified as a regularization parameter of 1 and a kernel coefficient of 0.001, resulting in an accuracy of 0.72 and an F1 score of 0.58. The random forest model performed best with a maximum tree depth of 20 and 50 trees, yielding an accuracy of 0.64 and an F1 score of 0.13. In the KNN model, the highest performance was achieved with five nearest neighbors and greater weight assigned to closer neighbors, producing an accuracy of 0.58 and an F1 score of 0.48. The naïve Bayes model achieved an accuracy of 0.47 and an F1 score of 0.39. The gradient boosting model demonstrated optimal performance with a learning rate of 0.1 and 100 boosting stages, achieving an accuracy of 0.78 and an F1 score of 0.64. For the decision tree model, the best configuration included a maximum tree depth of 20 and a minimum of five samples per node split, resulting in an accuracy of 0.67 and an F1 score of 0.54. The AdaBoost model performed optimally with a learning rate of 0.01 and 50 weak learners, achieving an accuracy of 0.56 and an F1 score of 0.50. The extra trees model yielded its best results with a maximum tree depth of 10 and a minimum of five samples per leaf, achieving an accuracy of 0.67 and an F1 score of 0.14. Lastly, the easyensemble model recorded an accuracy of 0.64 and an F1 score of 0.32. In conclusion, by applying the ADASYN oversampling technique, the gradient boosting model continued to achieve the highest accuracy and F1 score (Table 4).

**Figure 1:**
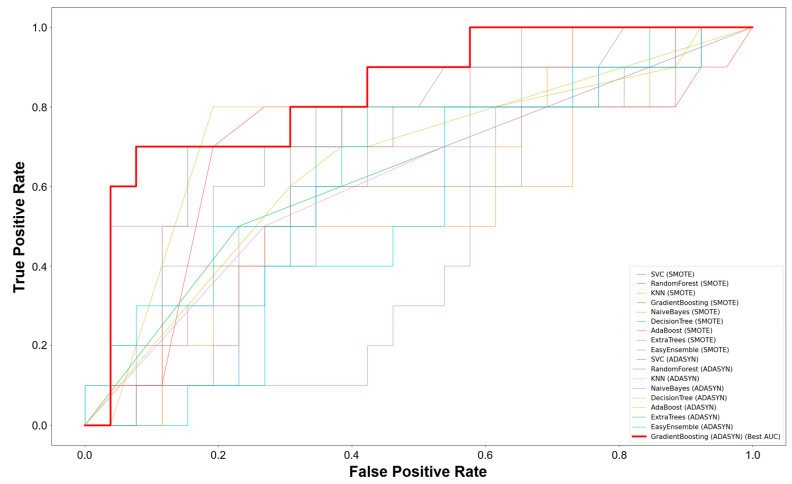
ROC curves for each model and technique. The gradient boosting model using the ADASYN technique showed the highest ROC AUC at 0.82. SVC, support vector classifier; KNN, K-nearest neighbors; SMOTE, synthetic minority over-sampling technique; and ADASYN, adaptive synthetic sampling method.

The ROC AUC scores demonstrated that the SVC model with SMOTE achieved a value of 0.69, while both the random forest and KNN models recorded a score of 0.62. The naïve Bayes model showed a score of 0.54, the gradient boosting model reached 0.75, the decision tree model scored 0.52, AdaBoost achieved 0.72, extra trees recorded 0.55, and easyensemble scored 0.68. When using the ADASYN technique, the SVC model scored 0.66, random forest also recorded 0.66, KNN scored 0.67, naïve Bayes reached 0.62, gradient boosting achieved 0.82, decision tree scored 0.61, AdaBoost recorded 0.73, extra trees scored 0.48, and easyensemble reached 0.64 (Figure 1). These findings indicate that the gradient boosting model consistently outperformed the others with both SMOTE and ADASYN, particularly with ADASYN, demonstrating the highest accuracy, F1 score, and ROC AUC values.

**Figure 2:**
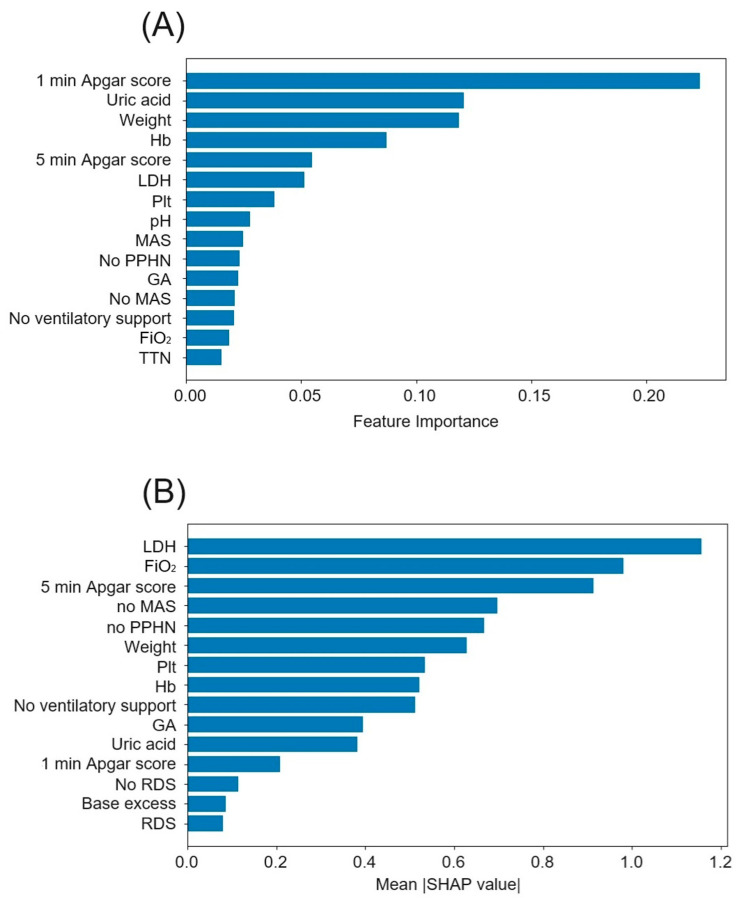
Top 15 features selected using gradient boosting. (**A**) Feature importance. The X-axis indicates the feature importance score. This score reflects how much each feature contributes to the model’s predictive accuracy. A higher score suggests that the feature plays a more significant role in making accurate predictions. (**B**) SHAP values. The X-axis indicates the mean |SHAP value|. This value represents the average magnitude of each feature’s impact on the model’s predictions across all samples. Higher SHAP values signify that the feature has a greater influence on the model’s output. Hb, hemoglobin; LDH, lactate dehydrogenase; Plt, platelet; MAS, meconium aspiration syndrome; PPHN, persistent pulmonary hypertension of the newborn; GA, gestational age; TTN, transient tachypnea of the newborn; RDS, respiratory distress syndrome; and SHAP, Shapley additive explanations.

The analysis of feature importance and SHAP values in predicting perinatal brain injury using the gradient boosting model identified crucial factors that enhance the model’s predictive accuracy (Figure 2). Feature importance (Figure 2A) displays the top 15 features based on their importance scores derived from the gradient boosting model. The 1 min Apgar score was identified as the most significant predictor, suggesting that how well a newborn manages the immediate transition from intrauterine to extrauterine life plays a critical role in the development of perinatal brain injury. In addition, the SHAP value analysis (Figure 2B) reflects each feature’s average impact on the model’s predictions. LDH was identified as having the highest SHAP values, indicating its critical influence on individual predictions.

## 5. Discussion

In our study, we aimed to develop a model that predicts perinatal brain injury using early postnatal data through machine learning. We optimized hyperparameters of multiple models, employing diverse approaches to manage imbalanced data. Consequently, we demonstrated that a machine learning model can effectively predict perinatal brain injury based on early postnatal data.

The gradient boosting model improves its performance iteratively by correcting errors from previous steps, focusing more on the predictions that were wrong in earlier models. This gradual improvement in accuracy is a key feature of gradient boosting. Furthermore, it offers the flexibility to fine-tune the learning process through hyperparameters such as the learning rate, which helps in avoiding overfitting [25]. Owing to these advantages, the gradient boosting model is considered to have demonstrated the highest accuracy, F1 score, and ROC AUC in our study. ADASYN and SMOTE are oversampling methods utilized to enhance the representation of minority class samples in imbalanced datasets [26,27]. In our study, these techniques were applied to address the clinical challenge where the number of confirmed brain injury cases from brain MRI results is inevitably higher than the number of non-injury cases. In comparison to SMOTE, ADASYN generates a greater number of synthetic samples for the minority class, particularly those that are nearer to or more integrated with majority class instances, thereby facilitating improved model learning in complex boundary regions. This enhanced the classification performance of the minority class by minimizing noise and overfitting [28]. The integration of ADASYN’s focus on challenging areas within the minority class, combined with the robust learning capabilities of gradient boosting, appears to have contributed to the exceptional performance demonstrated in our study.

In our study, a comparison between the no brain injury group and the brain injury group indicated that vaginal delivery was significantly linked to a higher incidence of brain injury compared to cesarean section. Although previous research proposed that cesarean section may be a risk factor for HIE [29] or perinatal stroke [30], the findings in this area remain inconclusive [31]. The 1 min and 5 min Apgar scores were lower in the brain injury group. In particular, the 1 min Apgar score was identified as the most influential predictor in the feature importance analysis. These results suggest that lower Apgar scores are associated with perinatal asphyxia, which is consistent with findings from previous studies [32,33]. The brain injury group also exhibited significantly lower pH levels, a result that was demonstrated in previous studies [34,35]. LDH was significantly higher in the brain injury group, and it was also identified as the most influential factor in the SHAP value analysis. This finding aligns with previous studies that indicated LDH levels as a good predictor of HIE [36]. Therapeutic hypothermia was more frequently observed in the brain injury group, which may be attributed to its use in managing severe cases of HIE.

We demonstrated that the application of the ADASYN technique to the gradient boosting model enabled accurate prediction of perinatal brain injury. We are confident that this machine learning model could play a pivotal role in identifying newborns who require brain MRI screening, facilitating timely interventions that may enhance long-term outcomes. Additionally, the model has the potential to reduce the number of unnecessary brain MRI scans, thereby contributing to a decrease in healthcare costs.

A limitation of our study is the relatively small sample size. Considering the imbalanced nature of the data and the inclusion of multiple predictive factors, expanding the dataset would likely enhance the model’s predictive accuracy and overall performance. Additionally, further study is needed to enhance accuracy through the development of diverse models, including those employing methodologies such as ANN [37].

## 6. Conclusions

Among the various models tested, the combination of ADASYN oversampling and the gradient boosting model demonstrated significant predictive capability for perinatal brain injury using early postnatal data. These findings suggest that machine learning has the potential to assist in the early identification of newborns who may benefit from targeted clinical interventions, ultimately improving long-term outcomes. In addition, our model could help reduce the reliance on unnecessary MRI scans, thereby contributing to more efficient healthcare resource allocation. Enhancing the model’s precision and effectiveness will require further development with a larger dataset to optimize its performance.

## Figures and Tables

**Table 1:** Variables used for predicting perinatal brain injury.

Variables	Sub Variables
Gestational age	
Birth weight	
Delivery method	Vaginal delivery, cesarean section
Apgar scores	1 min, 5 min
Maternal placental disease	Placental abruption, cord prolapse
Fetal heart rate abnormalities	Absent baseline fetal heart rate variability, recurrent late decelerations, recurrent variable decelerations, bradycardia
Neonatal symptoms	Hypotonia, apneic or periodic breathing, seizure
Blood pressure	Systolic, diastolic
Laboratory study	pH, pCO_2_, base excess, hemoglobin, platelet count, uric acid, albumin, LDH
Mode of ventilation	No ventilatory support, non-invasive ventilation, conventional ventilation, HFOV
FiO_2_	
Diagnosis	TTN, MAS, pneumothorax, RDS, PPHN
Therapeutic hypothermia †	

† Hypothermia treatment was initiated in response to an altered level of consciousness due to perinatal asphyxia [23]. FiO_2_, fraction of inspired oxygen; LDH, lactate dehydrogenase; HFOV, high-frequency oscillatory ventilation; TTN, transient tachypnea of the newborn; MAS, meconium aspiration syndrome; RDS, respiratory distress syndrome; and PPHN, persistent pulmonary hypertension of the newborn.

**Table 2:** Demographics of the no brain injury and brain injury groups.

	No Brain Injury	Brain Injury	*p* Value
Subjects, No.	137 (78)	39 (22)	
Sex			0.928
Male	88 (64.2)	26 (66.7)	
Female	49 (35.8)	13 (33.3)	
Gestational age (weeks ^+days^)	38 ^+0^ ± 1 ^+2^	38 ^+1^ ± 1 ^+3^	0.836
Birth weight (g)	3110 ± 533	2987 ± 472	0.137
Mode of delivery			0.01 *
Vaginal	31 (18.5)	17 (43.6)	
Cesarean section	106 (73)	22 (56.4)	
Apgar score			
1 min	7 ± 2	6 ± 3	0.001 *
5 min	9 ± 2	8 ± 3	0.048 *
Intrapartum complications			
Placental disease	6 (4)	1 (2.6)	1.0
Fetal heart rate abnormalities	12 (8.8)	5 (12.8)	0.538
Neonatal			
Hypotonia	12 (8.8)	6 (15.4)	0.238
Apneotic or periodic breathing	2 (2)	0 (0)	1.0
Seizure	32 (23.4)	6 (15.4)	0.397
Blood pressure			
Systolic (mmHg)	59 ± 7	58 ± 8	0.451
Diastolic (mmHg)	32 ± 7	32 ± 6	0.999
Laboratory study			
pH	7.22 ± 0.13	7.15 ± 0.15	0.004 *
pCO_2_ (mmHg)	59.2 ± 17.8	63.8 ± 20.4	0.185
Base excess (mmol/L)	−5.0 ± 5.4	−6.8 ± 6.9	0.156
Hemoglobin (g/dL)	17.3 ± 4.1	17.2 ± 2.3	0.755
Platelet count (/μL)	273,766 ± 73,407	256,333 ± 79,504	0.257
Uric acid (mg/dL)	6.0 ± 1.3	5.9 ± 1.5	0.546
Albumin (g/dL)	3.5 ± 0.4	3.6 ± 0.5	0.392
LDH (U/L)	695.3 ± 349.2	828.2 ± 329.7	0.005 *
Mode of ventilation			0.0860
No ventilatory support	39 (28.5)	5 (12.8)	
Noninvasive	27 (19.7)	9 (23.1)	
Conventional	48 (35.0)	16 (41.0)	
HFOV	23 (16.8)	9 (23.1)	
FiO_2_	0.35 ± 0.18	0.38 ± 0.24	0.429
Diagnosis			
TTN	32 (23.4)	7 (17.9)	0.473
MAS	10 (7.3)	6 (15.4)	0.126
Pneumothorax	5 (3.6)	4 (10.3)	0.111
RDS	25 (18.2)	6 (15.4)	0.814
PPHN	26 (19.0)	7 (17.9)	1.0
Therapeutic hypothermia	13 (9.5)	9 (23.1)	0.024 *

Values are expressed as mean ± standard deviation or number (%). FiO_2_, fraction of inspired oxygen; HFOV, high-frequency oscillatory ventilation; LDH, lactate dehydrogenase; TTN, transient tachypnea of the newborn; MAS, meconium aspiration syndrome; RDS, respiratory distress syndrome; and PPHN, persistent pulmonary hypertension of the newborn. * *p* < 0.05.

**Table 3:** Accuracy and F1 score of each model using the synthetic minority over-sampling technique.

Models	Accuracy (95% CI)	F1 Score (95% CI)
SVC	0.72 (0.61–0.81)	0.54 (0.35–0.55)
Randomf orest	0.69 (0.58–0.78)	0.15 (0.00–0.20)
KNN	0.64 (0.41–0.69)	0.52 (0.35–0.56)
Naive Bayes	0.50 (0.38–0.56)	0.44 (0.24–0.58)
Gradient boosting	0.72 (0.60–0.78)	0.58 (0.36–0.63)
Decision tree	0.56 (0.48–0.67)	0.27 (0.18–0.50)
AdaBoost	0.67 (0.41–0.69)	0.57 (0.34–0.63)
Extra trees	0.69 (0.58–0.78)	0.15 (0.00–0.20)
Easyensemble	0.72 (0.54–0.75)	0.44 (0.38–0.52)

SVC, support vector classifier; KNN, K-nearest neighbors.

**Table 4:** Accuracy and F1 score of each model using the adaptive synthetic sampling method.

Models	Accuracy (95% CI)	F1 score (95% CI)
SVC	0.72 (0.45–0.79)	0.58 (0.39–0.59)
Random forest	0.64 (0.55–0.74)	0.13 (0.00–0.23)
KNN	0.58 (0.42–0.62)	0.48 (0.31–0.61)
Naive Bayes	0.47 (0.36–0.56)	0.39 (0.24–0.58)
Gradient boosting	0.78 (0.64–0.83)	0.64 (0.49–0.66)
Decision tree	0.67 (0.54–0.72)	0.54 (0.38–0.56)
AdaBoost	0.56 (0.41–0.58)	0.50 (0.35–0.59)
Extra trees	0.67 (0.58–0.76)	0.14 (0.00–0.23)
Easyensemble	0.64 (0.57–0.75)	0.32 (0.10–0.47)

SVC, support vector classifier; KNN, K-nearest neighbors.

## Data Availability

The datasets generated and analyzed in the present study are available upon request from the corresponding author.
